# A dengue infection without bleeding manifestations in an adult with immune thrombocytopenic purpura

**DOI:** 10.1186/s41182-016-0036-3

**Published:** 2016-11-07

**Authors:** N. D. B. Ehelepola, M. B. K. Gunawardhana, T. N. Sudusinghe, S. K. D. Sooriyaarachchi, S. P. Manchanayake, K. L. R. Kalupahana

**Affiliations:** The Teaching (General) Hospital–Kandy, Kandy, Sri Lanka

**Keywords:** Dengue, Immune thrombocytopenic purpura, Platelet count, Prednisolone, Case report, Sri Lanka

## Abstract

**Background:**

Dengue is the most prevalent and fast spreading arboviral infection affecting people. No specific drug is available to treat dengue. Thrombocytopenia with potential of serious hemorrhages is one of the hall mark features of dengue. Immune thrombocytopenic purpura is an autoimmune disease causing thrombocytopenia. If a patient with that gets dengue, we expect severe thrombocytopenia with bleeding manifestations. Only a handful of such cases were reported before, and they were managed in different ways.

**Case presentation:**

A 30-year-old Sinhalese man recently diagnosed of immune thrombocytopenic purpura and on prednisolone was presented on the fourth day of fever, head ache, arthralgia, myalgia, and nausea. We started standard symptomatic dengue management and continued prednisolone. Dengue IgM and IgG antibody tests became positive. He was monitored by physical signs and serial full blood counts as the mainstay of monitoring. The patient never developed clinical bleeding manifestations and recovered.

**Conclusions:**

Considering the huge population at risk of dengue, generating more evidence on the topic and formulation of effective, simple guidelines to manage dengue in children and adults with immune thrombocytopenic purpura is going to be beneficial for many patients in the future.

## Background

Dengue is an emerging viral hemorrhagic fever with rare severe forms, transmitted by female *Aedes* mosquitoes. Management of clinical dengue is symptomatic because no specific antiviral drug is available [1–3]. Thrombocytopenia due to activation of complex immune mechanisms and direct dengue virus action on bone marrow is one of the hallmark features of dengue [2–5]. In severe dengue, disseminated intravascular coagulation can contribute to thrombocytopenia [5]. Platelet count is considered to correlate with the severity of dengue [2]. Serial platelet counts are a key laboratory investigation parameter in managing dengue patients. In some severe dengue cases, a combination of thrombocytopenia with other factors lead to life-threatening hemorrhages [2], hence a major concern to clinicians.

Immune thrombocytopenic purpura (ITP) also known as idiopathic thrombocytopenic purpura or immune thrombocytopenia is a hematological disorder resulting thrombocytopenia caused due to a primary or secondary autoimmune process [6]. It also can cause serious bleeding manifestations. Therefore, if a patient is diagnosed with ITP get infected with dengue, we, doctors, are more concerned and expect him/her to develop severe thrombocytopenia and bleeding manifestations. The current strongest estimate of the incidence of ITP is 3.3 per 100,000 adults per year [7]. Dengue incidence has dramatically risen around the world in recent decades [8]. Therefore, many patients with ITP are likely to get dengue infection at present and in the future, and it is important to be aware of the management of such cases. To our best knowledge, there are no guidelines regarding of management of such patients. We were surprised to read in one paper [4] that only four such cases were reported up to 2012 worldwide and none from Asia Pacific, the region with highest burden of dengue. We found one case report of a child from India [9]. Clinical course of ITP of children differs from adults [6]. Here, we report a case of an adult with recently diagnosed ITP getting dengue and recovered without developing any clinical bleeding manifestations. All four serotypes of dengue virus co-circulate in Sri Lanka, but the predominant serotype since 2009 is DENV1 [10]. With the above background, we think this case report will be interesting to many readers.

## Case presentation

A 30-year-old Sinhalese man was presented to us with history of high fever, head ache, generalized myalgia, arthralgia, loss of appetite, and nausea on the fourth day of the illness. He gave a history of digging a well a week before onset of fever and working at a rice paddy (exposure to leptospirosis). Leptospirosis was endemic, and a dengue epidemic was going on in his locality at that time. Six weeks ago, he developed a purpuric skin rash affecting all limbs with pruritus. After investigations including a bone marrow biopsy, a consultant hematologist has diagnosed ITP and started oral prednisolone and omeprazole. At the onset of his present illness, he has been on those drugs. He was also on topical clobetasol cream from a dermatology clinic for a patch of hypertrophic lichen planus at his left wrist. Apart from those, he was healthy.

On examination, he was not icteric or pale and had no skin rashes other than lichen planus rash. His pulse rate was 88/min, and blood pressure was 100/70 mmHg without a postural drop. Auscultation of lungs and abdominal examination was unremarkable. His oxygen saturation was 97 %.

Two most probable differential diagnoses were dengue and leptospirosis. Standard dengue fever management [1, 3] started, and in addition, intravenous crystalline penicillin was prescribed to cover leptospirosis. His skin sensitivity test for crystalline penicillin became positive. Hence, it was substituted with oral doxycycline, and prednisolone and omeprazole were continued.

Since his myalgia and arthralgia did not respond to oral paracetamol (acetaminophen) given *pro re nata*, a regular dose of oral tramadol was added. Oral calcium lactate was given from day 5 of the illness. On the seventh day of the illness, both dengue IgM and IgG were positive. Doxycycline was discontinued thereafter.

Table 1 summarizes his laboratory investigation results.

**Table 1:**
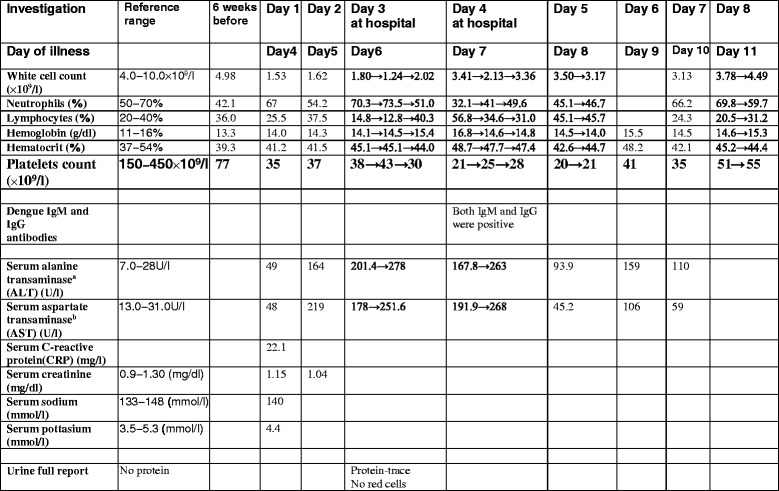
The summary of his laboratory investigation results

Platelet counts are captured in bold. Full (complete) blood count (FBC) done 6 weeks before is shown to get an idea of baseline (before starting prednisolone). No FBC immediately before onset of symptoms is available

^a^Serum alanine transaminase (ALT) is also known as serum glutamic pyruvic transaminase (SGPT)

^b^Serum aspartate transaminase (AST) is also known as serum glutamic oxaloacetic transaminase (SGOT)

His fever subsided after the sixth day of illness and never recurred. He developed marked flushing of the skin and conjunctival injection after the fifth day of illness that improved by the tenth day but never had any purpura or other bleeding manifestations; daily tourniquet tests were also negative during his hospital stay. He was sent home after 8 days and reassessed once and advised to continue to attend his hematology clinic. One week after discharge, his white cell count was 7.68 × 10^9^/l (neutrophils 72.7 % and lymphocytes 23.4 %), hemoglobin level was 14.9 g/dl with a hematocrit of 43.9 %, and the platelet count has risen to 97 × 10^9^/l. We did fasting blood sugar level test as well as he has been on steroids and it was also normal (4.1 mmol/l).

### Discussion

We discussed the relevant issues under four subheadings.

#### Diagnosis of dengue in this patient

A dengue epidemic was going on in his locality when he presented. His symptoms and signs were compatible with dengue. His laboratory reports also favor diagnosis of dengue (leucocytopenia, invertion of neutrophils to lymphocyte ratio on the seventh day of illness and again reversing, serial changes of his platelet count, serial changes of his hematocrit). Finally, positive dengue IgM and IgG tests confirmed the diagnosis. Infection with other flavoviruses can give false positives in IgM and IgG tests. Japanese encephalitis (JE) is the other flavovirus disease in Sri Lanka, and it is rare. JE is very rare in the central hill country where the patient lives and he has never traveled abroad and never got vaccinated against JE. Considering all, we can conclude this as a dengue case.

#### Comparison of this case with results of some past studies on thrombocytopenia and bleeding in dengue. Why he did not bleed?

Past studies from Sri Lanka and other countries show that in children with dengue, there is little correlation between platelet count and bleeding manifestations [4, 11, 12]. One study involving adult dengue patients demonstrated platelet counts <5 × 10^9^/l and hematocrit >50 % were significantly associated with bleeding manifestations. Another study suggested that bleeding occurred more often when platelet counts <20 × 10^9^/l that necessitate platelet transfusion [13]. However, one study done on adult dengue patients demonstrated no correlation between clinical bleeding and platelet count, and one third of their nonbleeding patients had counts <20 × 10^9^/l [14]. Before starting prednisolone, our patient had a purpuric rash although his platelet counts were higher. His platelet count did not drop below those risky levels, and the lowest was 20 × 10^9^/l in the morning of the eighth day of illness but quickly improved without any bleeding manifestations. The above evidences explain why he did not bleed.

#### What could have prevented severe thrombocytopenia, role of prednisolone

We wondered what prevented his platelet count dropping too low. The most likely possibility is he got a less severe form of dengue hemorrhagic fever (DHF). We speculate whether prednisolone he was on also might have helped him. Some of us and many other doctors known to us have used prednisolone, hydrocortisone, and methyl prednisolone to manage severe dengue, and in fact, some of us were treated with steroids as dengue patients in the past. There was a debate within the Sri Lankan medical fraternity of the use of steroids in dengue, and present dominant view is steroids are not beneficial. Complying with the latest national guidelines [3], we also have stopped using them except for especial cases like in this patient. Thrombocytopenia and capillary leakage (the corner stone of severe dengue), both are mainly resulting due to immune mechanisms. Temporary reversible disruption of the surface glycocalyx, a lining in the vascular endothelium that regulates microvascular filtration, is attributed to transient capillary leakage in dengue [15]. Immune mechanisms play a role in increased peripheral destruction of platelets and impairment of platelet functions in dengue [5]. Steroids blunt both innate and acquired immunity mechanisms responsible for these changes. There are evidences indicating that steroids modulate the function of the endothelial glycocalyx and may prevent damage to this layer [15]. We generally expect steroids to compromise immunity and favor viral replication. Viral load was correlated to severity of dengue in the past studies [2]. However, a laboratory study has shown that corticosteroids do not increase dengue viral replication in cell culture [15]. A randomized, placebo-controlled, blinded trial also showed that both low-dose and high-dose prednisolone therapy during viremic phase of dengue is not associated with prolongation of viremia or other adverse effects [15]. Nevertheless, this study did not show any benefits of prednisolone in preventing severe thrombocytopenia, shock or other complication of dengue as well [15]. Another placebo-controlled study employing sealed envelope randomization done in Sri Lanka showed low-dose intravenous dexamethasone was not effective in achieving a higher rise of platelet count in dengue infection [16]. According to the Cochrane review, there is insufficient evidence to evaluate the effects of corticosteroids in dengue at present [17]. However, the only other similar reported case who was also on steroids at the onset of fever and continued to receive steroids like our patient recovered after only having petechial hemorrhages [9]. His platelet count also did not fall <20 × 10^9^/l, and those authors mention that they contemplated to increase steroid dose in the case of bleeding or platelet counts dropping below that level [9]. Hence, steroids may be beneficial, at least unlikely to be harmful in treating dengue in diagnosed ITP patients when we consider above evidences. However, available evidences on the subject are inadequate to come to a reasonable conclusion.

#### Comparison of our management with that of other reported cases

An Indian boy who was also on steroids at the onset of dengue and continued to receive steroids like our patient recovered after only having petechial hemorrhages [9]. Other reported cases are from Americas. They were managed in different ways such as high doses of corticosteroids [18, 19], intravenous immunoglobulins (IVIG) [4], IVIG with steroids and platelet transfusion [19], and platelet and other blood product transfusion [20]. One 11-year-old Brazilian girl died [20]. Others recovered, and unlike our patient, most of them have previously undergone splenectomy. Some past studies indicate patients undergone splenectomy may be less susceptible to development of severe forms of dengue [4]. However, all of them had bleeding manifestations unlike our patient. Innate immunity and both humoral and cell-mediated immune processes are known to contribute to the pathogenesis of dengue hemorrhagic fever [2]. Unlike IVIG, steroids blunt all those immune processes. That might be a reason for no bleeding of our patient and minimal bleeding manifestations of the Indian boy. Nevertheless, some other reported patients received steroids also had bleeding, but steroids were started late in them [19].

It is interesting to note one Cuban patient was treated symptomatically, and there was a steep transient rise of platelet count much above her baseline level on the 15th day after onset of fever [21]. Our patient’s platelet count did not show such a rise. Persistence of thrombocytopenia (or onset of ITP) after dengue infections were also reported [22]. In contrast, our patient’s platelet count improved in days.

## Conclusions

Further studies involving larger numbers of patients would be helpful to determine whether steroids are beneficial and to formulate the best ways to manage dengue in adults and children with ITP. There is an acute need for such studies because 3.9 billion people, in 128 countries, are at risk of infection with dengue viruses at present [8].

## Abbreviations

ALT: Alanine transaminase
AST: Aspartate transaminase
DHF: Dengue hemorrhagic fever
ITP: Immune thrombocytopenic purpura
IVIG: Intravenous immunoglobulins
JE: Japanese encephalitis
